# A tale of two TROP-2 antibody-drug conjugates: a comparative saga of datopotamab deruxtecan and sacituzumab govitecan

**DOI:** 10.1093/oncolo/oyaf251

**Published:** 2025-08-06

**Authors:** Sanad Alhushki, Sameer Deshmukh, Benjamin Levy, Aakash Desai

**Affiliations:** Division of Hematology and Oncology, Department of Medicine, University of Alabama, Birmingham, AL, United States; Division of Hematology and Oncology, Department of Medicine, University of Alabama, Birmingham, AL, United States; Division of Hematology and Oncology, Department of Medicine, Johns Hopkins University, Baltimore, MD, United States; Division of Hematology and Oncology, Department of Medicine, University of Alabama, Birmingham, AL, United States

In the past decade, targeted therapies have transformed the treatment paradigm for oncogene-driven metastatic non–small cell lung cancer (NSCLC), while tumors without actionable genomic alterations typically receive chemotherapy combined with immunotherapy.^1^ However, the limited success of subsequent therapies underscores the need for new approaches. Antibody-drug conjugates (ADCs), which target specific tumor proteins and deliver cytotoxic agents directly to cancer cells, have emerged as an exciting option.^2^ Several ADCs targeting a variety of proteins are currently under development and have shown promise in this context. TROP-2, a common target overexpressed in many solid tumors including NSCLC,^3^ has been linked to worse outcomes, making it a critical focus for ADC development.^4^ Currently, two ADCs are in advanced stages of development in this space: Datopotamab Deruxtecan (Dato-DXd) and Sacituzumab Govitecan (SG). Both target TROP-2 but differ in their constructs and mechanisms.^5^ Dato-DXd comprises a monoclonal antibody linked to a topoisomerase inhibitor, allowing precise delivery of its chemotherapeutic payload to TROP-2-expressing cancer cells through a 4:1 drug-to-antibody (DAR) ratio.^6^ SG uses a similar antibody linked to a different cytotoxic payload, SN-38, which inhibits DNA replication through an optimized DAR of 8:1.^7^ Beyond Dato-DXd and SG, other TROP-2–targeting ADCs such as sacituzumab tirumotecan, temuratrexant-based conjugates and additional preclinical candidates are currently under investigation, underscoring the dynamic and competitive nature of this therapeutic space. While both Dato-Dxd and SG have been studied in second-line settings for the treatment of NSCLC, it is unclear how these agents compare to each other. Given that there is no randomized clinical trial comparing efficacy of these agents, we aimed to understand their comparative efficacy.

The individual patient data (IPD) from Kaplan-Meier (KM) survival curves (IPDfromKM) method offers a robust and flexible approach ideal for comparing outcomes across different therapies.^8^ We reconstructed individual patient data (IPD) from published KM curves using the IPDfromKM method, a validated, automated tool for digitizing and estimating time-to-event data. The process involves two key stages: (1) extraction of survival coordinates from KM plots, and (2) reconstruction of IPD using reported numbers at risk, censoring, and events. Coordinate data—representing time and survival probabilities—are preprocessed to ensure temporal ordering and monotonicity. These inputs are then iteratively modeled to estimate event distributions. We applied this method via a Shiny-based interface to digitize survival data from the TROPION-Lung01 (Dato-DXd) and EVOKE-01 (SG) trials, allowing for cross-trial comparison using harmonized survival endpoints.^9^ This methodological application ensures a high level of data fidelity and enhances our capacity to perform comparative analyses across different therapeutic interventions.

The TROPION-PanTumor01 trial initially showed activity of Dato-DXd across multiple tumor types, where it demonstrated notable efficacy in NSCLC.^10^ It observed an overall response rate (ORR) of approximately 25% across various NSCLC dose levels, with a median duration of response around 10 months, which supported further evaluation of this agent in NSCLC. Consequently, the TROPION-Lung01 phase III trial compared Dato-DXd to docetaxel in advanced NSCLC.^11^ In this trial, Dato-DXd demonstrated a progression-free survival (PFS) hazard ratio (HR) of 0.75, suggesting a 25% reduction in risk of disease progression or death compared to docetaxel. Median PFS (mPFS) was 4.4 months with Dato-DXd versus 3.7 months with docetaxel. Particularly in non-squamous NSCLC, Dato-DXd achieved a mPFS of 5.6 months, surpassing the 3.7 months with docetaxel, and a more favorable HR of 0.63. Conversely, in squamous NSCLC, Dato-DXd was less effective, showing a potential harm with an HR of 1.38 and shorter mPFS of 2.8 months compared to 3.9 months for docetaxel. Despite this modest improvement in PFS, no significant difference was notable in median overall survival (mOS) (12.9 vs 11.8 months; HR = 0.94 (0.78-1.14, p=.530).^11^

SG was first assessed in the IMMU-132-01 trial for NSCLC within a broader cohort of epithelial cancers, showing an overall response rate of 17% with noted gastrointestinal and cytopenic adverse effects.^12^ In the phase III EVOKE-01 trial, SG was compared against docetaxel in stage IV NSCLC after prior therapies. Although the primary endpoint of OS did not show a statistically significant improvement, with an HR of 0.84, SG demonstrated a median OS of 11.1 months versus 9.8 months for docetaxel. Notably, in patients nonresponsive to anti-PD-1/PD-L1 treatments, SG showed a median OS of 11.8 months compared to 8.3 months for docetaxel, indicating a potential benefit in this subgroup.^9^

**Figure 1.**
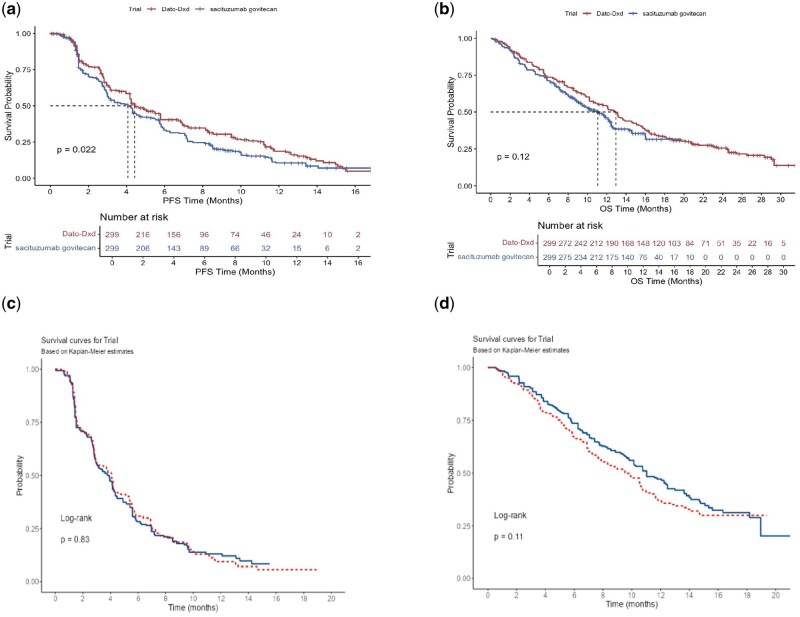
Reconstructed Kaplan-Meier (KM) survival curves using IPDfromKM from published phase III trials evaluating Datopotamab Deruxtecan (Dato-DXd) and Sacituzumab Govitecan (SG) in previously treated advanced non–small cell lung cancer (NSCLC). (a) Progression-free survival (PFS) for Dato-DXd vs SG. (b) Overall survival (OS) for Dato-DXd vs SG. (c) PFS for docetaxel control arms in TROPION-Lung01 and EVOKE-01. (d) OS for docetaxel control arms in TROPION-Lung01 and EVOKE-01. Curves were reconstructed using IPDfromKM based on digitized KM plots and trial-reported numbers at risk. Red, docetaxel from EVOKE-01; blue, docetaxel from TROPION-Lung 01.

In our comparative analysis of Dato-DXd and SG in the second-line treatment of NSCLC, the outcomes indicated distinct differences in efficacy. Dato-DXd showed a modest increase in efficacy with a mPFS of 4.4 months, compared to 4.1 months for SG, and a PFS HR of 0.81 (0.67-0.97, p = .022) (Figure 1a). Furthermore, Dato-DXd demonstrated superior OS with a median survival of 13.0 months compared to 11.1 months for SG 0.85 (0.69-1.05, p = .127); however, this was not statistically significant (Figure 1b). While these results show marginal benefit with Dato-DXd compared to SG, our applied framework highlights the potential for such computational modeling methods to provide additional insights from published clinical trials that may aid in clinical decision making and utilization of therapies (Table 1).

**Table 1.** Comparison of Dato-Dxd vs SG.

	Dato-Dxd vs SG	TROPION-Lung01	EVOKE-01
mPFS	4.42 vs 4.07	4.4 vs 3.7	4.1 vs 3.9
HR	0.81 (0.67-0.97, *P* = .022)	0.75 (0.61-0.91)	0.92 (0.77-1.11)
mOS	13.0 vs 11.1	12.4 vs 11.0	11.1 vs 9.8
HR	0.85 (0.69-1.05, *P* = .127)	0.92 (0.72-1.13)	0.84 (0.68-1.04)

Abbreviations: Dato-DXd, Datopotamab Deruxtecan; SG, Sacituzumab Govitecan; mPFS, median progression-free survival; HR, hazard ratio; mOS, median overall survival.

To better contextualize our findings, we also reconstructed survival curves for the docetaxel control arms from both the TROPION-Lung01 and EVOKE-01 trials using the IPDfromKM method. This comparison revealed a mPFS of 3.84 months (95% CI: 3.02–4.15) in TROPION-Lung01 vs 4.09 months (95% CI: 2.99–4.25) in EVOKE-01, with no statistically significant difference (HR 0.98; 95% CI: 0.81–1.18; p = .837). For OS, the docetaxel median OS was 11.0 months (95% CI: 10.1–12.5) in TROPION and 9.6 months (95% CI: 7.9–10.6) in EVOKE, also without statistical difference (HR 1.19; 95% CI: 0.96–1.47; p = .108). These results suggest comparable control arms, supporting the interpretability of our indirect efficacy comparisons between Dato-DXd and SG.

Our analysis of the clinical trials for Dato-DXd (TROPION-Lung01) and SG (EVOKE-01) paints a naunced picture of potential improvements in treatment outcomes without clear evidence of OS benefit. While Dato-DXd shows a marginal PFS benefit, particularly in non-squamous NSCLC, and SG shows promise in PD-1/PD-L1 resistant patients, neither agent convincingly demonstrated an OS benefit that is often crucial for FDA approval. In addition to efficacy, safety profiles are crucial in distinguishing between therapeutic options. SG was associated with higher rates of grade ≥3 gastrointestinal adverse events and neutropenia, which were reflected in EVOKE-01 as leading causes of dose modifications. Conversely, Dato-DXd in TROPION-Lung01 presented with notable stomatitis and ocular toxicity but lower hematologic toxicity. These differing toxicity profiles may inform clinical decision-making based on patient-specific risk factors and tolerability considerations. (Table 2)

**Table 2.** Comparative treatment-related adverse events (TRAE) in TROPION-Lung01 and EVOKE-01 trials. (Values are *n* (%). Grade ≥3 adverse events are shown in parentheses on the same row. Dato-DXd data from TROPION-Lung01 (*N* = 297); SG data from EVOKE-01 (*N* = 296). “–” indicates not reported in primary publication.)

Adverse event	Dato-DXd (*N* = 297) *n* (%)	SG (*N* = 296) *n* (%)
Any TRAE	260 (87.5) [Grade ≥3: 76 (25.6)]	295 (99.7) [Grade ≥3: 197 (66.6)]
Stomatitis/oral mucositis	141 (47.5) (20 [6.7])	39 (13.2) (3 [1.0])
Nausea	101 (34.0) (7 [2.4])	123 (41.6) (5 [1.7])
Diarrhea	30 (10.1) (4 [1.3])	156 (52.7) (31 [10.5])
Fatigue	–	168 (56.8) (37 [12.5])
Alopecia	95 (32.0) (0)	128 (43.2) (2 [0.7])
Decreased appetite	68 (22.9) (1 [0.3])	78 (26.4) (7 [2.4])
Anemia	44 (14.8) (12 [4.0])	119 (40.2) (19 [6.4])
Neutropenia	14 (4.7) (2 [0.7])	111 (37.5) (73 [24.7])
Leukopenia	9 (3.0) (0)	38 (12.8) (15 [5.1])
Febrile neutropenia	–	23 (7.8) (23 [7.8])
Asthenia	56 (18.9) (8 [2.7])	–
Peripheral neuropathy	–	11 (3.7) (0)
ILD/Pneumonitis (adjudicated)	26 (8.8) (11 [3.7])	–
Treatment-related AEs leading to discontinuation	24 (8.1)	20 (6.8)
Treatment-related deaths	3 (1.0)	10 (3.4)

Abbreviations: Dato-DXd, Datopotamab Deruxtecan; SG, Sacituzumab Govitecan; AEs, adverse events.

Recent exploratory analysis from the TROPION-Lung01 showed TROP2 expression was predictive of clinical outcomes in patients treated with Dato-DXd.^13^ However, prospective validation of these results in a dedicated clinical trial setting will be required to incorporate this into clinical practice. Similarly, the EVOKE-01 trial showed promising results in patients previously unresponsive to PD-1/PD-L1 inhibitors in a post hoc analysis.^14^ However, given the lack of standardized definition of anti-PD-1/PD-L1 resistance, varied mechanisms of resistance in this group, and an overall lack of OS benefit, these findings are not yet clinically meaningful.

Computational models can help navigate the complexities of therapeutic evaluation in NSCLC. By facilitating a deeper understanding of kinetic profiles and patient response variability, it provides a sophisticated analytical framework that can inform both the design and interpretation of clinical trials. We hope that its integration into future research will enable enhanced predictive accuracy and support decision-making processes in oncology. Using IPDfromKM to analyze subsets of patients with distinct biomarkers across different trials may allow discovery of detailed therapeutic responses within biomarker-enriched patient populations, facilitating a more tailored approach in select populations. Furthermore, if the model highlights meaningful differences in drug efficacy, it could lay the groundwork for a prospective comparative study between drugs. Such a study would not only address a clinically relevant question but would also serve to validate the model. In anticipation of multiple, nuanced therapeutic strategies entering the competitive treatment-naïve and second-line treatment settings, the role of innovative comparative analytical methods like IPDfromKM becomes increasingly crucial.

It is important to note that while IPDfromKM allows for robust extraction and reconstruction of survival data, discrepancies in the survival curves’ original presentation can introduce biases. The method assumes consistent reporting of patient numbers at risk across the curves, which might not always be the case. Variations in trial design, endpoint definitions, and patient populations can limit the direct comparability of reconstructed IPDs.

Despite therapeutic advancements, the oncology community remains in a state of flux, characterized by competing strategies and an abundance of clinical questions that current trials are unable to fully answer. This study focused primarily on TROP-2 ADC trials, while, in the first-line EGFR space, multiple approaches based on trials like FLAURA2 (Osimertinib plus chemotherapy) and MARIPOSA (amivantamab and lazertinib) highlight the ongoing challenges there. The potential coupling of these novel modalities with real-world data will provide a more comprehensive understanding of treatment impacts in the future, but meanwhile, the IPDfromKM model has shown considerable promise in retrospective analyses. Future prospective trials could be strategically designed to rigorously compare therapeutic responses, providing statistically sound evidence to enhance its validity and utility in clinical settings. By bridging the gap between theoretical prediction and real-world efficacy, this approach would solidify such computational models as a future cornerstone of precision oncology.

In conclusion, while both Dato-DXd and SG offer promising directions, their path to regulatory approval and clinical utility is fraught with challenges. The current landscape of NSCLC treatment demands innovative approaches and broader collaborations to address pressing clinical questions. As we continue to integrate AI tools in oncology, IPDfromKM and other such computational models will emerge as an instrumental component of this technological evolution. While not yet on par with fully developed AI systems, the IPDfromKM model represents a significant step toward sophisticated, data-driven decision-making in clinical research, underscoring its potential to evolve alongside AI advancements. The future of methodologies like IPDfromKM holds promise but requires further exploration and validation to solidify their role in oncology.
